# Neck–Shoulder Region Training for Chronic Headache in Women: A Randomized Controlled Trial

**DOI:** 10.1177/02692155231170687

**Published:** 2023-04-25

**Authors:** Marjo Rinne, Sanna Garam, Katriina Kukkonen-Harjula, Kari Tokola, Arja Häkkinen, Jari Ylinen, Riku Nikander

**Affiliations:** 1 UKK Institute for Health Promotion Research, Tampere, Finland; 2 School of Rehabilitation and Examination, Metropolia University of Applied Sciences, Helsinki, Finland; 3 Faculty of Sport and Health Sciences, 4168 University of Jyväskylä, Jyväskylä, Finland; 4 Faculty of Medicine and Health Technology, 7840 Tampere University, Tampere, Finland; 5 Department of Physical Medicine and Rehabilitation, Central Hospital of Central Finland and Central Finland Wellbeing Services County, Jyväskylä, Finland

**Keywords:** headache, exercise, training, strength, neck disability, randomized controlled trial, women

## Abstract

**Objectives:**

We investigated whether a specific exercise program for the neck–shoulder region reduces headache intensity, frequency, and duration, and how it influences neck disability among women with chronic headache compared to a control group.

**Design:**

Two-center randomized controlled trial.

**Subjects:**

116 working-age women.

**Intervention:**

The exercise group (n = 57) performed a home-based program with six progressive exercise modules, over 6 months. The control group (n = 59) underwent six placebo-dosed transcutaneous electrical nerve stimulation sessions. Both groups performed stretching exercises.

**Main measures:**

The primary outcome was pain intensity of headache, assessed using the Numeric Pain Rating Scale. Secondary outcomes were frequency and duration of weekly headaches, and neck disability assessed using the Neck Disability Index. Generalized linear mixed models were used.

**Results:**

Mean pain intensity at baseline was 4.7 (95% CI 4.4 to 5.0) in the exercise group and 4.8 (4.5 to 5.1) in the control group. After 6 months the decrease was slight with no between-group difference. Headache frequency decreased from 4.5 (3.9 to 5.1) to 2.4 (1.8 to 3.0) days/week in the exercise group, and from 4.4 (3.6 to 5.1) to 3.0 (2.4 to 3.6) in the control group (between-group *p* = 0.017). Headache duration decreased in both groups, with no between-group difference. Greater improvement in the Neck Disability Index was found in the exercise group (between-group change −1.6 [95% CI −3.1 to −0.2] points).

**Conclusion:**

The progressive exercise program almost halved headache frequency. The exercise program could be recommended as one treatment option for women with chronic headache.

## Introduction

Headache cause considerable disability, 52% of the world population suffer from various types of headache.^1,2^ Headaches due to neck pain and sore muscles in the neck–shoulder region are commonly diagnosed as tension-type or cervicogenic headaches.^3^ Chronic headache, presenting as tension-type headache episodes and/or migraine attacks, is defined occurring at least 15 days per month, prevailing over 3 months, and chronic migraine features at least 8 days a month.^3^ All headache types are more prevalent in women than in men, and chronic headache and migraine are the most common types.^2^

To manage chronic cervicogenic headache, tension-type headache and migraine, strength^4–6^ and aerobic training,^4–6^ stretching,^4,5^ relaxation,^4,5^ manual treatment,^4,5,7^ and cognitive therapy^4^ have been applied as non-pharmacologic options.^4–7^ In review and meta-analysis by Luedtke et al.^5^ trigger-point therapy, manual therapy, and combinations of physical and psychological treatments were reported to markedly reduce tension-type headache pain intensity, headaches duration, and the number of migraine attacks, and manual therapy reduce cervicogenic headache pain intensity and frequency. The level of evidence was reported as low. Specific therapeutic exercises have been proposed as treatment options for cervicogenic headache.^7,8^ Few randomized controlled trials on chronic headache have included large numbers of outpatients and used a single-component approach, such as a progressive exercise program.^8–10^

The aim of this randomized trial in working-age women with chronic headache was to investigate whether a specific progressive therapeutic exercise program for the neck–shoulder region is effective in reducing the intensity, frequency, and duration of headache in comparison to the control group receiving a placebo dose of transcutaneous electrical nerve stimulation. We also investigated whether the exercise program would reduce neck disability and improve neck function.

## Methods

We conducted a 6-month randomized controlled trial at two centers (UKK Institute, Tampere, Finland, and Metropolia University of Applied Sciences, Helsinki, Finland) from October 2012 to June 2013. The trial design has been published elsewhere.^11^ The trial was approved by the Ethics Committee of Pirkanmaa Hospital District, Finland in January 2012 (Code R12006) and was prospectively registered at ClinicalTrials.gov (NCT01664585). The study was conducted according to the guidelines of the Declaration of Helsinki. All participants signed a written informed consent form. They were informed that they would be participating in a physical therapy study for in which they would be randomized either to the neck–shoulder region training group or to the group administered transcutaneous electrical nerve stimulation treatment, for 6 months.

**Figure 1.**
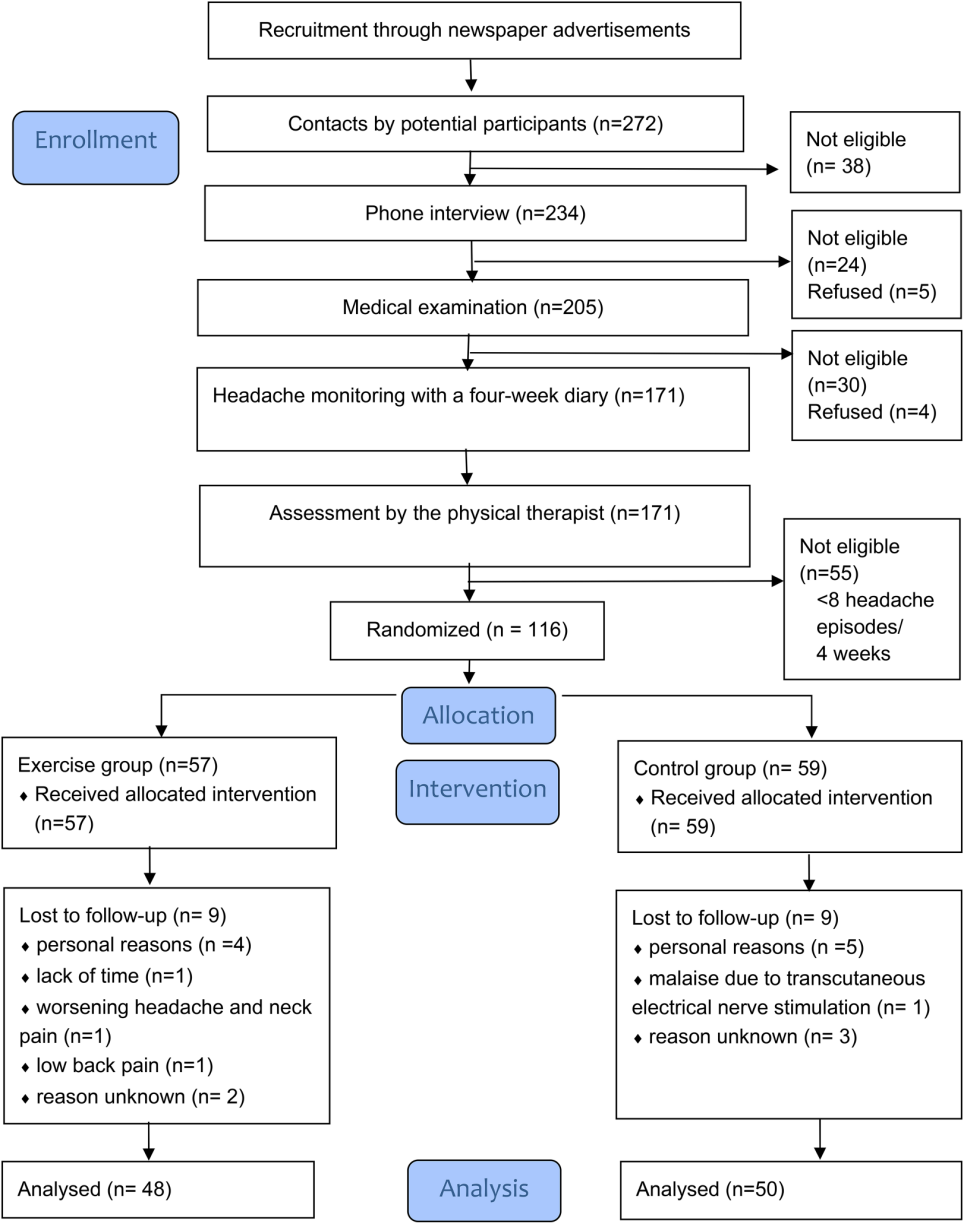
Flowchart of the trial.

We recruited women aged 18–60 years with chronic headache by using newspaper advertising. The flowchart of the study is presented in Figure 1. Eligibility was assessed in three screening process phases. The inclusion criteria^11^ were: working women, report of at least 8 days of headache (with intensity scoring of 40 mm or more on the Visual Analogue Scale)^12,13^ during 4 consecutive weeks, and a score of 56 points or more on the Headache Impact Test^TM^ ^14^ (scale 36 to 78 points). The exclusion criteria^11^ were: depressive symptoms according to the Beck Depression Inventory (>19 of 63 points),^15^ alcohol abuse (Alcohol Use Disorders Identification Test^16^ >6 points), whiplash injury or other neck injury, previously in health care verified severe degenerative changes in the cervical vertebrae or intervertebral disks, including disk prolapse, habitual physical activity three or more times weekly, and during the previous month prior to baseline: changed headache medication, received manual therapy or other physical therapy treatments because of headache or changed bifocals.^11^ Three study physicians examined volunteers to assess their eligibility. Eighteen (16%) of the randomized participants were lost before the end of the 6-month intervention. There were two adverse effects in the exercise group: one participant reported worsening headache and neck pain, and another experienced backache. One participant in the control group experienced considerable malaise related to transcutaneous electrical nerve stimulation. All three participants discontinued the trial.

The randomization schedule was generated by a statistician who was not involved in the recruitment of the participants. Block randomization with block sizes of 2, 4, 6, and 8 was used. The randomization sequence involved stratification according to the two study centers. The group assignments were placed in consecutively numbered, sealed, opaque envelopes. After a woman met the eligibility criteria during the baseline-assessment visit to the physical therapist, she opened the next available envelope of her stratum.

A sample size of 49 women in each group was calculated to detect a decrease of 20 mm or more on the Visual Analogue Scale (pain intensity), over 6 months, with 90% power and an alpha of 0.05.^17^ A dropout rate of 25% was expected.^11^ Of the 272 women volunteering for the study, 116 were eligible; 57 women were assigned to the exercise group and 59 to the control group.

The 6-month progressive therapeutic exercise program in the exercise group consisted of six modules; the first three included low-load exercises for the neck–shoulder region, and the remaining three specific strengthening exercises for the neck and upper body. Three stretching exercises were included from the third module onward.^11^ The modules were customized to a progressive program (Appendix 1) according to training protocols based on Jull et al.^7^ and Ylinen et al.^8,17^ Two experienced physical therapists instructed the participants at the beginning of each module, the first two modules were supervised individually, and the remaining four in small groups. The participants continued training at home supported by an instructional booklet and videos in Finnish (link: http://bit.ly/2CW3HQ7 [Videos 1–7]). The participants were advised to perform the exercises six times per week during the first four modules, for about the first 3 months, and four times per week during the latter two, for about the latter 3 months.^11^ In the first two modules exercises took approximately 15 min to perform and in the latter four modules exercises were performed in approximately 30 min.

The control group underwent six individual 45-min sessions consisting of placebo-dosed treatment with 20 min of transcutaneous electrical nerve stimulation (less than 10 mA, phase width 150 ms, and 100 Hz constant frequency [Chattanooga Intelect Advanced device 2765CS, Chattanooga Group Inc., Hixson, Tennessee, US]) once a month for 6 months, rather than weekly as suggested.^18^ Treatment parameters and the placement of the electrodes^11^ differed from that used for people with chronic tension-type headache.^18^ From the third session the control group received instructions to perform the same three stretching exercises three times a week as the exercise group.^11^ Both groups were advised to maintain their habitual physical activity throughout the 6-month period.

Pain intensity (primary outcome), and frequency and duration (secondary outcomes) of headache episodes were captured using weekly diaries. The participants reported the intensity of each headache episode by using a segmented Numeric Pain Rating Scale (0–10 integers) which is a validated version of the Visual Analogue Scale,^13^ the frequency of headache episodes, the duration of each episode, and the use of headache-related medication in the diaries.^11^ Weekly exercise diaries were used to record completed program exercise sessions as well as other physical exercises.^11^

At baseline and at 6 months, neck disability was assessed with the Neck Disability Index^19^ and headache impact on daily life with the Headache Impact Test^TM^.^14^ The physical therapist measured isometric endurance of the flexor and extensor muscles of the cervical spine with submaximal tests,^20^ and the range of active movements in the cervical spine in the sitting position with a goniometer (Myrin Goniometer^®^, Model 927034, Medema Physio AB, Solna, Sweden).

Participants’ age, education, and occupation were enquired at baseline. At baseline and at 6 months, they were asked about medication, menstruation status, sleep, depressive symptoms (using the Beck Depression Inventory^15^), work ability (using the Work Ability Index^21^), habitual physical activity, physical strain at work, and smoking habits. Daytime physical activity and sedentary time were monitored using a three-dimensional waist-worn accelerometer (Hookie AM20, Traxmeet Ltd, Espoo, Finland) for 1 week at baseline and at 6 months.^11^ Baseline characteristics of the two groups were compared using the independent-samples’ *t*-test for comparison of means, and the non-parametric Mann–Whitney *U* test was used when data were not normally distributed. Fisher's exact test was used for the comparison of proportions.

Between-group differences in the intensity, frequency, and duration of headache episodes based on diaries were analyzed according to the intention-to-treat principle. Data were analyzed in 4-week periods at three time points (baseline, 3 months, and 6 months) with generalized linear mixed models with confounding variables as covariates (age, type of work, smoking, use of headache medication, Work Ability Index score, type of headache, hormonal medication, menstruation status). In the case of count data (e.g., frequency of headache) Poisson or negative binomial regression was used. Prior to analyses, covariates and time were decided as random effects. The potential effect of the study center as a covariate was checked afterward (intra-class correlation coefficient 0.07% for the Numeric Pain Rating Scale), and the study center was excluded from the final models. Neck disability, neck muscle endurance, and neck range of motion were analyzed by analysis of covariance. Odds ratio was used in the analysis of endurance of neck extensors. Effect size was calculated by using Cohen's *d*. All the analyses were performed using IBM SPSS software (version 24, IBM Corp., Armonk, NY, USA).

Before the group assignment was revealed, statistical analyses were conducted with masked identifiers and the statistician was blinded to the group assignment. Complete analyses and blinded interpretation^22^ are provided as Supplement A1. The first and last author vouched for the accuracy and completeness of the reported data and the analyses. Furthermore, two external reviewers scrutinized the blinded interpretation.

## Results

Baseline characteristics are presented in Table 1. The mean age of the participants was 44.2 years (SD 9.3), 55 (47%) participants had tension-type headache, cerviocranial syndrome or cervical spondylosis with headache, and 61 (53%) were diagnosed as having migraine or migraine with cervical pain^23^.

**Table 1.** Baseline characteristics of the participants in the exercise group and control group.

	Exercise group (*n* = 57)	Control group (*n* = 59)	*p*
Age (y), mean (SD)	45.8 (8.6)	42.6 (9.7)	.06*
Headache type *n* (%)			.85^†^
Tension-type headache	18 (31.6)	21 (35.6)	
Cervicocranial syndrome	9 (15.8)	4 (6.8)	
Cervical spondylosis with headache	1 (1.8)	2 (3.4)	
Migraine and migraine with cervical pain	29 (50.9)	32 (54.2)	
Education, *n* (%)			.53^†^
High school	9 (15.8)	6 (10.2)	
Vocational education	35 (61.4)	35 (59.3)	
University	13 (22.8)	18 (30.5)	
Married or cohabitating, *n* (%)	47 (82.5)	39 (66.1)	.06^†^
Full-time employment, *n* (%)	48 (84.2)	50 (84.7)	.96^†^
Physical strain at work, *n* (%)**			.40^†^
Mostly sitting or physically light	31 (54.4)	36 (61.0)	
Moving/walking	22 (38.5)	22 (37.3)	
Strenuous	4 (7.0)	1 (1.7)	
Menopausal, *n* (%)	27 (47.4)	29 (49.2)	.86^†^
Nonsmokers, *n* (%)	50 (87.7)	53 (89.8)	.78^†^
Depressive symptom score^||^, mean (SD)	6.0 (5.2)	7.8 (5.9)	.08^‡^
Duration of leisure physical activity by intensity classes, time (h:min/day), mean (SD)^#^			
Light	3:24 (1:04)	2:58 (0:48)	.03*
Moderate	0:42 (0:18)	0:42 (0:23)	.98*
Vigorous	0:03 (0:07)	0:03 (0:05)	.78^‡^
Sedentary time (h:min/day), mean (SD)**			
Working day	7:44 (3:01)	8:07 (2:51)	.48*
Weekend or day off	5:01 (2:20)	5:46 (2:39)	.14*
Daily steps (n)^#^	7589 (2352)	7185 (2687)	.42*

Between-group differences (p-values) were analyzed using an independent-samples t- test*, the Mann–Whitney *U* test^†^, and Fisher's exact test^‡^.

**Subjective estimation; ^||^Beck's Depression Inventory^15^ range of scores from 0 to 63; ^#^3D accelerometer Hookie AM20 (Traxmeet Ltd., Espoo, Finland).

Forty-one women (72%) in the exercise group participated in all six prescribed supervised sessions, and 24 (41%) in the control group participated in all six placebo transcutaneous electrical nerve stimulation sessions. In the exercise group, the mean weekly number of completed home training sessions was 4.1 (SD 1.2) of the prescribed six during the first 3 months, and 2.8 (SD 1.4) of the prescribed four during the latter 3 months. In the control group, the mean weekly number of completed stretching exercises was 2.2 (SD 0.6) during first 3 months, and 0.5 (SD 0.3) during the latter 3 months.

**Figure 2.**
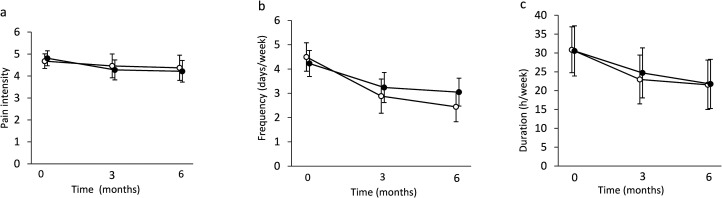
Pain intensity (a), frequency (b), and duration (c) of weekly headaches at baseline, after 3 months, and after 6 months in the exercise 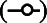 group and in the control group 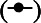. Means (95% CI).

At baseline mean headache intensity was 4.7 (95% confidence intervals, CI 4.4 to 5.0) in the exercise group and 4.8 (4.5 to 5.1) in the control group. Over the 6-month intervention, the mean intensity decreased by −0.6 (SD 1.3) in the exercise group and by −0.4 (SD 1.3) in the control group, with no between-group difference (group*time interaction, *p* = 0.66) (Figure 2(a)). The effect size *d* was 0.17.

At baseline, the mean frequency of headache days/week was 4.5 (95% CI 3.9 to 5.1) in the exercise group and 4.4 (3.6 to 5.1) in the control group (Figure 2(b)). Over 6 months the frequency decreased more in the exercise group (−2.2 (SD 2.3)) when compared to the control group (−1.2 (SD 2.9)) (*p* = 0.017 between-group), and the effect size *d* was 0.53.

At baseline, the mean weekly duration of headache episodes was 30.8 (95% CI 24.7 to 36.9) hours/week in the exercise group and 30.5 (23.9 to 37.1) in the control group (Figure 2(c)). Over 6 months the decrease was 11.3 (SD 23.5) hours/week in the exercise group and 5.6 (SD 26.0) in the control group. There was no significant between-group difference (group*time interaction, *p* = 0.24) in the change in duration of headache episodes. The effect size *d* was 0.27.

Neck flexor endurance time increased by 22 s (95% CI 11 to 33, *p *< 0.0002) more in the exercise group compared to the control group (Table 2). At baseline, in the static neck extensor muscle test 72% of the exercise group and 79% of the control group achieved the maximum of 180 s. After 6 months 93% of the exercise group and 71% of the control group achieved the maximum (odds ratio 9.2; 95% CI 2.0 to 42.1). After 6 months the exercise group had significantly greater improvement in cervical spine rotation (between-group difference 8° [95% CI 1 to 15]). The Neck Disability Index declined 1.6 (95% CI −3.1 to −0.2) points more in the exercise group than in the control group, and the Headache Impact Test declined −2.0 (95% CI −4.3 to 0.2), respectively.

**Table 2.** Neck function, Neck Disability Index, and Headache Impact Test^TM^ at baseline and after the 6-month trial in the exercise group and in the control group. Means (SD) or mean changes (95% confidence intervals).

	Exercise group		Control group		Between-group changes after 6 months
	Baseline	6 months	Baseline	6 months	
	(n = 57)	(n = 48)	(n = 59)	(n = 50)	
Neck muscle endurance/static flexion test^a^, sec	54.3 (48.8)	86.8 (55.6)	48.2 (39.5)	53.3 (46.0)	21.8 (10.5 to 33.1)
Range of motion, degrees					
Flexion	53.0 (10.0)	56.0 (8.7)	56.5 (10.9)	61.2 (10.3)	−3.2 (−6.9 to 0.4)
Lateral flexion^b^	70.3 (19.9)	78.9 (10.1)	73.5 (14.4)	79.0 (12.2)	2.5 (−1.6 to 6.7)
Rotation^b^	121.8 (20.6)	131.4 (17.6)	127.2 (20.3)	126.9 (21.0)	7.8 (1.0 to 14.5)
Neck Disability Index^c^	11.7 (4.4)	8.8 (5.3)	10.6 (4.5)	9.0 (3.8)	−1.6 (−3.1 to −0.2)
Headache Impact Test^d^	63.0 (3.3)	55.7 (6.7)	63 (3.6)	57.6 (5.5)	−2.0 (−4.3 to 0.2)

^a^ Maximum hold time of the static test position up to 180 s.

^b^ Values are the sum of the right and left sides.

^c^ Scores can vary from 0 to 50.

^d^ Scores can vary from 36 to 78 points.

## Discussion

This randomized trial on working-age women with chronic headache showed that after 6 months of progressive training for the neck–shoulder region, headache pain intensity, our primary outcome, decreased slightly in the exercise group, but also in the control group, which received a placebo dose of transcutaneous electrical nerve stimulation. However, after the 6 months of training, weekly headaches occurred less frequently in the exercise group than in the control group. The duration of headache episodes decreased in both groups.

To the best of our knowledge, our randomized trial is the largest clinical headache trial to apply a specific progressive exercise program for the neck–shoulder region. We succeeded in retaining the majority of the participants throughout the 6-month intervention. Before randomization, volunteers were carefully screened for eligibility in three phases.^11^ Approximately half the participants in each group had different types of headache with neck pain and tension-type headache, and the other half had migraine with cervical pain.

The progression of exercise intensity in the exercise group was slow. Low-load exercises were prescribed for the first three modules about 10 weeks and strengthening exercises for the neck–shoulder region were prescribed for the remaining weeks of the intervention. The exercise program was well tolerated, confirming the findings of previous studies indicating that women with chronic headache tolerate training of the neck–shoulder region.^8–10^ There were two adverse effects, one participant dropped out of the exercise program due to worsened headache and neck pain and another due to back pain.

We investigated effects of a specific neck–shoulder exercise program instead of aerobic exercises^6,24,25^ to alleviate headache. The present study substantiates the findings of previous studies indicating that women with chronic headaches including migraine also tolerate well-strengthening exercises for the neck–shoulder region.^8–10^ However, La Touche et al.^24^ showed that aerobic exercises can decrease migraine pain intensity, frequency, and duration as well as increase quality of life. Lemmens et al.^25^ in their meta-analysis provided similar results concerning the frequency of migraine. However the quality of evidence was moderate.^25^

In our study, the there was no change in pain intensity after the progressive program including strengthening exercises. Varangot-Reille et al.^26^ found in their meta-analysis that strengthening exercises had moderate effect on pain intensity in patients with tension-type headache.^26^ Ylinen et al.^8^ investigated the efficacy of specific neck muscle strength training, and endurance training on cervicogenic headache intensity in women during 12 months, compared to the stretching exercises. Both training programs reduced headache intensity more than stretching. The study supports the use of specific neck strength training in the treatment of women with headache.^8^ Madsen et al.^10^ compared 10 weeks of strength training of the shoulder region in people with tension-type headache to correction of ergonomy and posture. There was no between-group difference in the frequency, duration, or intensity of headache pain or in the use of medication.^9^ Andersen et al.^10^ investigated the effect of 20-week strength training for the neck–shoulder area in adults with headache. Specific strength training of the neck–shoulder area for 1 h weekly reduced headache frequency and intensity by about 50% in the exercise group when compared to the control condition.^10^

Headaches occurred less frequently in the exercise group compared to controls. The mean reduction in weekly headache days was 47% in the exercise group. Such a reduction in headache frequency could have a great impact on daily life. The observed reduction was also clinically relevant.^9,24,27^ The control group exhibited a 32% reduction in the mean frequency of weekly headache days. This might have been due to the attention given individually by the physical therapist during the placebo transcutaneous electrical nerve stimulation sessions, and adherence to home stretching exercises. Nevertheless, stretching has been found to be more effective when combined with strength training than when performed alone among women with cervicogenic headache.^8^ Duration of headache episodes also decreased in both groups which heightens the impact of decreased headache frequency.

The Neck Disability Index improved more in the exercise group when compared to the control group, but the change did not reach clinical relevance.^28^ Static muscle endurance time of the neck flexor muscles increased by 60% in the exercise group and by 11% in the control group. Ylinen et al.^17^ found similar changes in isometric neck strength in an exercise trial on chronic neck pain. In addition, the range of cervical spine rotation increased more in the exercise group than in the control group. Neck strengthening exercises have been shown to improve neck rotation in female office workers with chronic neck pain.^17^

The strengths of this rigorously conducted randomized controlled trial are large number of participants with chronic headache, low proportion of withdrawals, and blinded data analyses. A further strength is the use of a placebo treatment instead of a waiting list. However, our study has limitations. At the time the study design was planned, the best evidence preferred headache intensity as the primary outcome.^29^ More recent research has proposed headache frequency as the primary outcome of trials.^26,27^ Another weakness is that our physical therapists were not blinded when assessing outcomes.

Our therapeutic exercise program for the neck–shoulder region decreased headache frequency, but not headache intensity when compared to the control group, which received placebo-dosed transcutaneous electrical nerve stimulation. The participants had different kinds of chronic headaches, including migraine with recurrent headache episodes. The reason for the minor effects of the intervention in terms of headache intensity is unknown. However, we can speculate that since the participants had migraine among other headache types, headache intensity may have stayed quite high during headache episodes, even if the frequency of headache episodes decreased. This can have an impact on the mean weekly headache intensity. We conclude that this progressive exercise program for the neck–shoulder region is feasible and safe to perform, and could be used as a treatment option for women with chronic headache. We suggest that further therapeutic exercise research could focus on different subgroups of headaches, as mechanisms vary by headache types.

**Clinical messages**

Working-age women with chronic headache were instructed on a strengthening exercise program, which was compared to placebo-dosed transcutaneous electrical nerve stimulation.
After 6 months headache pain intensity decreased slightly in both treatment groups.
The exercise program for the neck–shoulder region almost halved weekly headache days and was safe to perform.

## Supplemental Material

sj-docx-2-cre-10.1177_02692155231170687 - Supplemental material for Neck–Shoulder Region Training for Chronic Headache in Women: A Randomized Controlled Trial Click here for additional data file. Supplemental material, sj-docx-2-cre-10.1177_02692155231170687 for Neck–Shoulder Region Training for Chronic Headache in Women: A Randomized Controlled Trial by Marjo Rinne, Sanna Garam and Katriina Kukkonen-Harjula, Kari Tokola, Arja Häkkinen, Jari Ylinen, Riku Nikander in Clinical Rehabilitation

sj-pdf-1-cre-10.1177_02692155231170687 - Supplemental material for Neck–Shoulder Region Training for Chronic Headache in Women: A Randomized Controlled Trial Click here for additional data file. Supplemental material, sj-pdf-1-cre-10.1177_02692155231170687 for Neck–Shoulder Region Training for Chronic Headache in Women: A Randomized Controlled Trial by Marjo Rinne, Sanna Garam and Katriina Kukkonen-Harjula, Kari Tokola, Arja Häkkinen, Jari Ylinen, Riku Nikander in Clinical Rehabilitation
