# Supplementary material for: Neck–Shoulder Region Training for Chronic Headache in Women: A Randomized Controlled Trial
Source: Clin Rehabil. 2023 Apr 25;37(10):1322–31. doi: 10.1177/02692155231170687 (PMC10426253; doi:10.1177/02692155231170687)
Supplement: sj-docx-2-cre-10.1177_02692155231170687 - Supplemental material for Neck–Shoulder Region Training for Chronic Headache in Women: A Randomized Controlled Trial [file sj-docx-2-cre-10.1177_02692155231170687.docx]

**Appendix 1. Procedures for Therapeutic Exercises and Stretching Exercises**

1. **Therapeutic Exercises**

The 6-month therapeutic exercise regimen consisted of six periods with progressive training modules.

All therapeutic exercises used in the study are described below according to the aim, instructions to perform, and dosage. It is pointed out in which periods and Modules they are included.

**Exercises in Modules I and II, duration 2 weeks each**

**1. Retraction of shoulder blades, prone**

Aim: To train the muscles in the posterior scapular region.

Instructions: Prone, a folded towel under the forehead. Hold your neck in a neutral position, and arms relaxed by sides. Pull the shoulder blades towards the thoracic spine, upper arms follow the movement. Return to the starting position.

Dosage:

In Module I: Repeat the movement 5 times with a 5-second hold at the peak of the movement. Between each repetition rest for 5 seconds.

In Module II: Repeat 10 times with a 10-second hold at the peak of the movement. Between each repetitions rest for 10 seconds.

# 2. Exercises for deep neck flexors - craniocervical flexion

Aim: To train deep flexor muscles of the neck.

Instructions: Supine, with a folded towel under the upper neck. Hold your neck in a neutral position, and arms by sides. Nod your chin towards your chest without lifting the head. Return to the starting position.

Dosage:

In Module I: Repeat the movement 5 times with a 5-second hold at the peak of the movement, and between each repetition rest for 5 seconds.

In Module II: Repeat the movement 10 times with a 10-second hold at the peak of the movement, and between each repetition rest for 10 seconds.

**3. Head lift, prone**

Aim: To strengthen the muscles in the back of neck, and to control the posture of the cervical spine.

Instructions: Prone, a folded towel under the forehead. Your neck should be relaxed in a neutral position, elbows flexed, and hands flat on the floor beside your shoulders.

Lift the head and shoulders by the muscles of the neck and upper back. Keep the cervical spine in a neutral position, and hold the position throughout the movement. The movement should be slow and controlled, including the return to the starting position.

Dosage:

In Module I: Repeat the movement 5 times with a 5-second hold. Between each repetition rest for 5 seconds.

In Module II: Repeat the movement 10 times with 10-second hold. Between each repetition rest for 10 seconds.

**4. Isometric exercises for the flexor and extensor muscles of the cervical spine**

Aim: To learn to activate and contract the flexors and extensors of the cervical spine isometrically, and to stabilize the position of the head and neck properly while leaning the trunk forward.

**4a. Isometric cervical flexion**

Instructions: Sitting up straight, with your shoulders relaxed and hands on your laps. Place the palm of one hand against the forehead.

Push your head forward against your palm, and resist with your hand. The anterior muscles of the neck contract isometrically. Maintain the of head and neck unchanged, without hunching your shoulders. Relax after each muscle contraction.

Dosage:

In Module II: Isometric muscle contraction for 5 seconds. Perform 1 to 3 times.

**4b. Isometric cervical extension**

Instructions: Sitting up straight, with your shoulders relaxed and hands on your laps. Place the palm of one hand against the back of the head.

Push your head backward, and resist with your hand. The posterior muscles of the neck contract isometrically. Maintain the position of head and neck unchanged, without hunching shoulders. Relax after each muscle contraction.

Dosage:

In Module II: Isometric muscle contraction for 5 seconds. Perform 1 to 3 times.

**Exercises in Modules from III to IV, duration 6 weeks each**

**5. Exercises for the flexor and extensor muscles of the cervical spine with a rubber band**

Aim: To learn to activate and contract the extensors of the cervical spine to stabilize the neutral position of the head and neck while leaning the trunk forward.

**5a. Specific exercise for cervical flexion with a rubber band**

Instructions: Sit up straight, with an elastic rubber band around your forehead, and the other end fixed to a solid object (e.g., a door handle). Shoulders and arms are relaxed, and hands on your laps. Keep the spine straight, and lean your torso forward from hips until the rubber band around the forehead begins to resist maximally the movement. Activate the anterior muscles of neck isometrically without lifting the chin, and maintain the same posture in the neck and head throughout the movement. Return to the starting position.

Dosage:

In Module III: Repeat 10 times using yellow rubber band as resistance, and perform 2 sets. In the second set target for a rating of perceived exertion (RPE)* between 10 to 12.

In Module IV: Repeat 10 to 15 times using red rubber band as resistance, and perform 3 sets, with a 1-minute rest between the sets. In the third set, target for perceived exertion between 12 to 14 RPE*.

In Module V: Repeat 15 times using the red rubber band, and perform 3 sets with a 1-minute rest between sets. In the third set, target for perceived exertion between 12 to 14 RPE *.

**5b. Specific exercise for cervical extension with a rubber band**

Instructions: Sit up straight, with an elastic rubber band around the back of your head, and the other end fixed to a solid object (e.g., a door handle). Shoulders are relaxed, and hands on your laps. Keep the spine straight, and lean your torso backward from hips until the rubber band against the back of the head begins to resist maximally the movement. Activate the posterior muscles of the neck isometrically, without lifting the chin, and maintain the same posture in the neck and head throughout the movement.

Dosage:

In Module III: Repeat 10 times using the red rubber band as resistance, and perform 2 sets. In the second set, target for perceived exertion is between 10 to 12 RPE *.

In Module IV: Repeat 10 to 15 times using the red rubber band as resistance, and perform 3 sets with a 1-minute rest between sets. In the third set target for perceived exertion between 12 to 14 RPE*.

Module V: Repeat 15 times using the red rubber band as resistance, and perform 3 sets with a 1-minute rest between sets. In the third set target for perceived exertion between 12 to 14 RPE*.

**6. Reciprocal flexion with extended arms**

Aim: To control the muscles and posture of the cervical spine and the upper trunk during reciprocal movements with extended arms.

Instructions: Stand up straight, with light dumbbells or weights in hands. Move the upper limbs upward and down by turns. Keep the arms fully extended throughout the movement. Maintain for perceived exertion between head and neck in a neutral position, and keep for perceived exertion between shoulders relaxed.

Dosage:

In Modules III, IV, and VI: Exercise for 30 seconds per 1 set. Perform 2 sets, with a rest between the sets. In the second set target for perceived exertion between 10 to 12 RPE*.

**7. One-arm extension on all fours**

Aim: To control the muscles and posture of the cervical spine and upper trunk while elevating the extended arm.

Instructions: While kneeling on all fours on a mat, ensure the proper alignment of the spine, neck and torso. Your knees should be vertically beneath hips, and arms vertically beneath shoulders, with hands flat against the mat. Push slightly upward with arms, but avoid overextending elbows. Elevate the arm extended up to the level of shoulder height. Hold this position, and return back to all fours. Repeat with the other arm.

Dosage:

In Module IV: Hold the arm elevated in the extended position for 10 seconds. Repeat 5 times with both arms.

In Module V: Hold the arm elevated in the extended position for 10 seconds. Repeat 5 times with both arms. Perform 1 to 3 sets.

In Module VI: Hold the arm elevated in the extended position for 10 seconds. Repeat 5 times with both arms for 3 sets.

**8. Supine head lift**

Aim: Dynamic strengthening exercise for the neck flexor muscles.

Instructions: Supine, with a folded towel under the upper neck. With your arms by side and neck relaxed in a neutral position. Nod and lift the head toward the chest. Return to the starting position with the chin pulled in.

Dosage:

In Module V: 5 to 8 repetitions.

In Module VI: 10 to 15 repetitions.

1. **Stretching exercises**

The stretching exercises were instructed for both study groups starting from the Module III. Every stretch is instructed to be performed on both sides with 3 repetitions with a 30-second hold.

**1. Stretch in the cervical lateral flexion**

Sitting upright, with good posture. Slowly rotate the head downward, with the chin pointing toward the armpit. Avoid lifting your shoulders up during the stretch. Hold the stretch for 20 seconds, then slowly raise your head to the neutral position, and repeat the stretch on the other side.

**2. Stretch of the anterior neck muscles**

Sitting upright, with good posture. Keep the shoulders down and relaxed. Place the other hand on the opposite shoulder. Slowly tilt the head to the other side and backward, look diagonally to the ceiling.

Hold the stretch for 20 seconds, and return the head back to the neutral position. Repeat the stretch on the other side.

**3. Stretch of the pectoralis major muscle**

A standing position with the body sideways to a wall. Press your forearm against the wall, with your elbow bent at 90 degrees. Turn the body away from the wall until the feeling of the stress during the stretch across the chest is mild-to-moderate. Hold the stretch for 20 seconds and repeat it 3 times with both arms.

*Borg G. Psychophysical bases of perceived exertion. *Med Sci Sports Exerc.* 1982;14: 377–381.
