# Supplementary material for: Neck–Shoulder Region Training for Chronic Headache in Women: A Randomized Controlled Trial
Source: Clin Rehabil. 2023 Apr 25;37(10):1322–31. doi: 10.1177/02692155231170687 (PMC10426253; doi:10.1177/02692155231170687)
Supplement: sj-pdf-1-cre-10.1177_02692155231170687 - Supplemental material for Neck–Shoulder Region Training for Chronic Headache in Women: A Randomized Controlled Trial [file sj-pdf-1-cre-10.1177_02692155231170687.pdf]

Supplement A1.

THERAPEUTIC EXERCISE TRAINING TO REDUCE CHRONIC HEADACHE IN WORKING WOMEN

Blinded Data Analyses Statement of Interpretation

(ClinicalTrials.gov Protocol Registration, NCT01664585; Protocol article of the RCT: Rinne M, Garam S, Häkkinen A, Ylinen J, Kukkonen-Harjula K, Nikander R. Therapeutic Exercise Training to Reduce Chronic Headache in Working Women: Design of a Randomized Controlled Trial. Phys Ther 2015, in press)

Background

Our study comprises two groups, a therapeutic exercise training group (THEX) and a very low dose (comparable to placebo) Transcutaneous Electrical Nerve Stimulation (TENS) group. We use the placebo-dose TENS treatment as a comparison group to ensure an equal amount of attention and care from the caregivers (placebo effect) to try to avoid potential bias related to "meaning effect". Therapeutic exercise training has been shown to be effective for chronic neck pain in institutional rehabilitation interventions, and it has shown some potential with headache patients (see references at the end of this document as supplementary material), even though "the current level of evidence is low and whether and which physiotherapy approach is effective" is still unknown (Luedtke et al. 2015).

Hypothesis

We assume the therapeutic exercise training including stretching exercises (THEX intervention group) offers a benefit to headache-suffering working women over the effect of placebo-dose TENS treatment including stretching exercises (control group).

Commitments for data analysis

1) To be deemed effective, the THEX intervention should provide a statistically significant benefit ($p < 0.05$) over the placebo-dose TENS treatment in the primary (average intensity per week; Visual Analogue Scale) and secondary (frequency; times per week, and duration; hours per week) outcomes of headache, assessed at 0, 3, and 6 months during the six-month RCT.

2) For the observed THEX effect to be deemed clinically relevant, THEX should also provide > 15 mm greater reduction in VAS intensity than TENS, and one headache attack less per week than TENS (based on the literature that is provided as supplementary material at the end of this document).

Statistical analysis plan

a) For the primary and secondary outcomes, intention-to-treat (ITT) is the primary data analysis. Ancillary analyses will also be carried out. Efficacy analysis will be performed to evaluate whether there are subgroups that benefit from the program: for example, whether patients with high training adherence benefit more than patients with low training adherence, or whether some types of headache, e.g., migraine, either substantially benefit from the program or the program increases their headache intensity, frequency, and/or duration (a potential adverse effect of the intervention).

b) Muscle strength and neck and shoulder flexibility will be also analyzed, but only as explanatory outcomes to provide support (theoretical basis and mechanisms) for possible significant findings in the analysis of the primary and secondary outcomes presented above. This means that between-group differences in the changes of disability, fear avoidance beliefs, work ability, quality of life, fatigue, depressive symptoms, ergonomics at work, and leisure time physical activity will be assessed in the interpretation of the findings. However, these analyses are used only as background information and explanatory outcomes, and to measure and interpret a potential intervention effect throughout the musculoskeletal system and in mental health in addition to social participation in society according to the International Classification of Functioning, Disability and Health (ICF), a framework for measuring health and disability. Since we utilize several outcomes in our analyses, we also use Benjamini & Hoecberg's False Discovery Rate to avoid type 1 statistical error.

c) Ancillary analyses will be also performed using three Generalized Linear Mixed Models (GLMM) for each outcome: 1) unadjusted, 2) adjusted by age, type of work (office vs. other), smoking (no vs. yes), and 3) adjusted by age, type of work, smoking, use of headache medication (weekly frequency), work ability index, type of headache (migraine vs. cervicogenic headache), hormone therapy (no vs. yes), menstruation status (normal, irregular, or perimenopausal/postmenopausal). Data will be analyzed as longitudinal with 3 time points (baseline, 3 months and 6 months). Outcomes with only 2 measurements will be analyzed with Generalized Linear Models (GLM) with the same models as presented above.

Analysis protocol

Groups were blinded and coded as Group E and Group S by MR (who was not blinded to the data) who then delivered the data to the statistician (KT) and last author (RN). The last author prepared the first draft of the two interpretations of the blinded results based on the intention to-treat analysis approach.

Blinded review of the data: A writing committee meeting (May 27, 2015)

The Writing Committee of the THERAPEUTIC EXERCISE TRAINING TO REDUCE CHRONIC HEADACHE IN WORKING WOMEN trial (undersigned) discussed the two interpretations of the results on the basis of a blinded review (Group E compared to Group S) with one

approach assuming that Group E was the therapeutic exercise training + stretching (THEX) group, and the other assuming that Group E was the placebo-dose TENS + stretching (TENS) group. The first author (MR) participated in the meeting as a secretary but did not participate in preparing the interpretation of the results.

Based on these theoretical commitments, our interpretation of the findings are as follows:

a) If the THEX group is found superior to the TENS group during the six-month intervention (clinically relevant improvement in headache pain vs. placebo, as defined above), the study is applicable to the patient group studied, but its generalizability remains unknown.

b) If the THEX group is not found superior to the TENS group, the study suggests that therapeutic exercise training with six booster sessions to support home-based training during the six-month intervention period does not work for outpatients suffering from chronic cervicogenic headache and migraine in a pragmatic real-life situation. This could be due to an insufficient training amount and intensity in the THEX group or an increased leisure time physical activity amount in the TENS group, all of which will be analyzed from the explanatory outcomes. If this is the case, an efficacy design would be warranted to fully assess the efficacy of THEX (under optimal circumstances). However, given the high external validity, such a finding (of no effectiveness) would strongly suggest that THEX does not work in ordinary outpatient settings in primary healthcare. This assumption requires that the training adherence in the intervention group has been sufficient to achieve a decline in headache intensity.

c) If the TENS group is found superior to the THEX group during the six-month intervention, we will conclude that the specific therapeutic exercise training (THEX) does not provide any benefit to the outpatient group suffering from chronic cervicogenic and tension-type headache with migraine compared to very low-dose (placebo) Transcutaneous Electrical Nerve Stimulation (TENS). Thus, the specific therapeutic exercise training program should not be recommended to the patient group studied.

Based on the blinded results, our interpretation of the findings is:

Interpretation 1. The THEX group was Group E and thus it was superior to the TENS Group (Group S):

We found that the six-month therapeutic exercise training (THEX) decreased headache frequency clinically relevantly compared to the low-dose Transcutaneous Electrical Nerve Stimulation treatment (TENS). In detail, participants in the THEX group reduced their number of weekly headaches from 4.6 to 2.4, while the TENS group participants reduced their number of weekly headaches from 4.3 to 3.1 ($p=0.017$). The effect size for reduction of headache frequency was 0.53. THEX did not reduce headache intensity and duration. The training did not affect the use of headache medications and overall quality of life. Thus, because of the reduced number of headache episodes, therapeutic training (THEX) can be recommended as an effective treatment for outpatients with headaches, such as patients with cervicogenic and tension-type headache with migraine.

Interpretation 2. The TENS group was Group E and thus it was superior to THEX Group (Group S):

We found that the (placebo) Transcutaneous Electrical Nerve Stimulation (TENS) did not reduce headache intensity and duration. However, it reduced headache frequency clinically relevantly when compared to the therapeutic exercise training (THEX). In detail, the placebo group reduced their number of weekly headaches from 4.6 to 2.4, while the THEX group reduced their number of weekly headaches from 4.3 to 3.1 (p=0.017). The effect size for the reduction of headache frequency was 0.53. The placebo TENS did not affect the use of headache medications and overall quality of life. However, placebo treatment cannot be recommended as a serious therapy for clinical practice.

Jan 8th 2016

Marjo Rinne, PhD, PT

Jan 14, 2016

Sanna Garam MSc, PT

Jan 11, 2016

Arja Häkkinen, PhD, PT

[illegible] 13, 20[illegible]

Jari Ylinen, MD, PhD

Jan 8, '16

Katriina Kukkonen-Harjula, MD, PhD

Jan 8th 2016

Riku Nikander, PhD, PT

8.1.2016

Kari Tokola, MSc

Appendix. Supplementary material.

1. Theoretical basis

It has been proposed that female gender, poor physical function and body posture among other things can predispose for neck pain and headache (Macfarlane et al. 2009). Analgesics and other medication should be recommended as short term but not long term treatment for chronic neck pain (A working group appointed by the Finnish Medical Society Duodecim. Neck pain. 2009. www.kaypahoito.fi/web/english, in Finnish, abstract in English). In migraine and tension-type of headaches, pharmaceutical therapy forms the basis of the treatment, however, little is known about the therapeutic exercise training and its effects (Busch and Gaul 2008, Racicki et al. 2013, Luedtke et al. 2015). Pharmaceutical treatment is also offered as a primary treatment for tension-type headache supported by various forms of physiotherapy methods as secondary treatments (Luedtke et al. 2015). Physiotherapy has been typically offered as the first option for cervicogenic headache. However, physiotherapy methods applied have varied greatly with these patients as well.

It has been shown that energy storage in muscle mitochondria is lower in patients suffering from chronic neck pain than in healthy individuals (Ylinen 2004). Moreover, local blood circulation inside muscles is reduced resulting in compromised oxygen transportation and overall metabolism. Also, muscle strength is lower in chronic neck pain patients compared to healthy individuals (Ylinen 2004). In addition, muscle atrophy and increased amount of fat tissue inside muscles has been observed in chronic neck patients compared to healthy individuals (Ylinen 2007).

Thus, principles of therapeutic exercise in successfully treating cervicogenic headache could also utilize therapeutic exercise and consist of low load and low intensity proprioceptive training at the early phase and specific high-load and high intensity exercises in the later phase of the program, or the former or the latter throughout the treatment period (Jull 2002, Busch et al. 2008, Fricton et al. 2009, Ylinen et al. 2010). On the other hand, current level of evidence to treat headache effectively suggest that the level of evidence is low. Moreover, there is not information on 'whether and which physiotherapy approach is effective' (Luedtke et al. 2015).

We have chosen a single component approach to treat tension-type and cervicogenic headache in addition to migraine because diagnoses of these patients seem to overlap considerably in clinical work. Thus, we utilize combination of low load proprioceptive training exercises and specific high-load high intensity neck muscle training exercises to reverse the above described catabolic/detrimental processes with the goal of achieving normal body posture movement control of the cervical spine, muscle metabolism and muscle structure.

This RCT is designed to address the feasibility of a single component physiotherapy approach in ordinary healthcare setting, i.e. whether therapeutic exercise training can work in real-life for typical outpatients with severe chronic headache. Aiming for a group representative of typical headache outpatients, we recruited patients with migraine, tension-type and cervicogenic headaches. Accordingly, we have chosen liberal eligibility criteria in recruiting, aiming for a group that would be representative for typical headache outpatients.

2. Key references for setting the magnitude of clinical relevance in terms of the primary outcome (favoring the active intervention group in all cases)

1. Ylinen et al. 2010: between-group-difference in the change of headache intensity was 10 to 15 mm (corresponding to 10-15%, including the whole scale) in VAS (intervention groups: endurance training vs. control group, and strength training vs. control group). NB. The intervention started with a short institutional rehabilitation period where training adherence can be well controlled.
2. Gross et al. 2015 (meta-analysis): between-group-difference in the change of pain intensity was less than 15 mm (corresponding to <15%, including the whole scale) in VAS (intervention groups: cervical stretch/range of motion exercises + cervical/scapulothoracic strengthening + static/dynamic cervical/shoulder stabilization vs. wait list). NB. The intervention included patients with all kinds of mechanical disorders with or without cervicogenic headache and radiculopathy.
3. Gram et al. 2014: between- group-difference in the change of headache pain scale was -1.1 (corresponding to 12% including whole scale) in a scale of 0 to 9 (intervention groups: supervised training group vs. reference group without exercise training). NB. Patients were office workers (outpatient study) and headache patients were included in this analysis.
4. Kääriäinen R 2014: Conclusion: Studies have shown that cervicogenic headaches and migraine interventions do not appear to reduce the intensity of the headache (Level of evidence B). In: The effectiveness of therapeutic exercise in the treatment of headaches. A systematic review and meta-analysis of randomized controlled trials. Physiotherapy Master `s Thesis University of Jyväskylä, Faculty of Sports Science, Department of Health Sciences. 2014. In Finnish with English abstract. open access, https://jyx.jyu.fi/dspace/bitstream/handle/123456789/43630/URN%3aNBN%3afi%3ajyu-201406041932.pdf?sequence=1
5. Luedtke et al. 2015 (meta-analysis): Reduction in tension-type headache pain intensity was 11mm (VAS 0-100mm) and cervicogenic headache 25mm (no reduction with migraine). Also, reduction in headache frequency indicated 1.3 days less headaches per month in tension-type headache and 22.4 hours less time without pain relief in migraine and 1.7 hours less headache per day in cervicogenic headache. However conclusion states exactly that: 'Results suggest a statistically significant reduction in the intensity, frequency and duration of migraine, tension-type headache and cervicogenic (CGH) headache. Pain reduction and reduction in CGH frequency do not reach clinically relevant effect sizes. Small sample sizes, inadequate use of headache classification and other methodological shortcomings reduce the confidence in these results. Methodologically sound, randomized controlled trials with adequate sample sizes are required to provide information on whether and which physiotherapy approach is effective. According to Grading of Recommendations Assessment, Development and Evaluation (GRADE), the current evidence is low' (Luedtke et al. 2015).

Figure 1. Effect of therapeutic exercise training on cervicogenic headache (Kääriäinen 2014).

Study or Subgroup	Control Mean	Control SD	Control Total	Exercise Mean	Exercise SD	Exercise Total	Weight	Std. Mean Difference IV, Random, 95% CI
Jull 2002 exercise	0.75	2.33	25	0.48	4.5	52	12.8%	0.07 [-0.41, 0.55]
Jull 2002 manip+ exerc.	0.75	2.33	25	0.47	3.72	49	12.7%	0.08 [-0.40, 0.57]
Kongsted 2007 exercise	0.7	0.59	77	0.4	0.59	140	16.9%	0.51 [0.22, 0.79]
Kongsted 2007 Head restraint	0.7	0.59	77	0.8	0.59	144	17.0%	-0.17 [-0.45, 0.11]
Scholten 2006	0.39	0.57	42	0.62	0.56	38	13.5%	-0.40 [-0.85, 0.04]
Ylinen 2010 endurance	0.65	0.67	32	0.37	0.72	59	13.7%	0.39 [-0.04, 0.83]
Ylinen 2010 strength	0.65	0.67	32	0.3	0.63	57	13.5%	0.54 [0.10, 0.98]
**Total (95% CI)**			**310**			**539**	**100.0%**	**0.15 [-0.13, 0.43]**

Heterogeneity: $Tau^2 = 0.10$; $Chi^2 = 21.59$, df = 6 (P = 0.001); $I^2 = 72\%$
Test for overall effect: Z = 1.04 (P = 0.30)

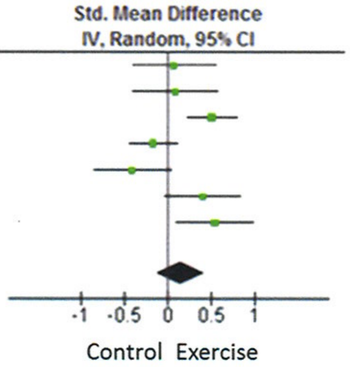

Figure 2. Effect of therapeutic exercise or relaxation on migraine (Kääriäinen 2014).

Study or Subgroup	Control Mean	Control SD	Control Total	Exercise Mean	Exercise SD	Exercise Total	Weight	Std. Mean Difference IV, Random, 95% CI
John 2007	1.02	0.12	33	0.63	0.14	32	24.6%	2.96 [2.24, 3.67]
Lemstra 2002	0.98	0.28	36	0.56	0.25	41	25.4%	1.57 [1.06, 2.09]
Varkey 2011 exercise	0.72	0.73	16	0.88	0.76	30	25.0%	-0.21 [-0.82, 0.40]
Varkey 2011 relaxation	0.72	0.73	16	0.88	0.74	30	25.0%	-0.21 [-0.82, 0.39]
**Total (95% CI)**			**101**			**133**	**100.0%**	**1.02 [-0.38, 2.42]**

Heterogeneity: $Tau^2 = 1.94$; $Chi^2 = 63.90$, df = 3 (P < 0.00001); $I^2 = 95\%$
Test for overall effect: Z = 1.43 (P = 0.15)

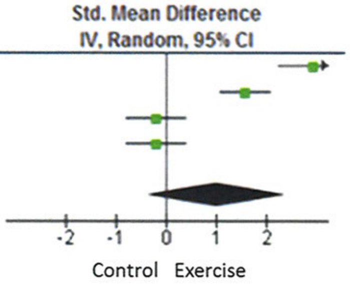

Figure 3. Effect of therapeutic exercise on tension neck (Kääriäinen 2014).

Study or Subgroup	Control Mean	Control SD	Control Total	Exercise Mean	Exercise SD	Exercise Total	Weight	Std. Mean Difference IV, Random, 95% CI
Söderberg 2011 exercise	0.66	1.91	9	0.67	1.95	19	50.0%	-0.01 [-0.80, 0.79]
Söderberg 2011 relaxation	0.66	1.91	9	0.6	1.23	19	50.0%	0.04 [-0.75, 0.83]
**Total (95% CI)**			**18**			**38**	**100.0%**	**0.02 [-0.54, 0.58]**

Heterogeneity: $Tau^2 = 0.00$; $Chi^2 = 0.01$, df = 1 (P = 0.94); $I^2 = 0\%$
Test for overall effect: Z = 0.06 (P = 0.95)

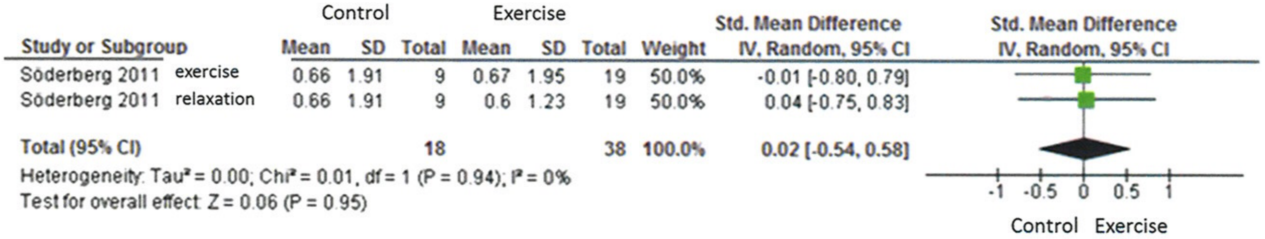

3. Summary of outcome variables and key statistical analyses (primary outcome and secondary outcomes)

	a) Power analysis performed based on this * b) Required sample	Statistical method	Output style	a) p-value ** b) effect size (Cohen's d)
**Headache intensity (VAS, 0 to 100 mm)**	**a) YES b) n=98**	**GLMM**	**Mean (SD) Change (95% CI)**	**a) 0.05 b) 0.2**
Headache frequency (number of headaches in a month)	a) NO	GLMM	Mean (SD) Change (95% CI)	a) 0.05 b) 0.2
Headache duration (hours per week)	a) NO	GLMM	Mean (SD) Change (95% CI)	a) 0.05 b) 0.2
Muscle Strength (according to Edmonson 2008 without extra weight)	a) NO	GLMM	Mean (SD) Change (95% CI)	a) 0.05 b) 0.2
Neck and shoulder Flexibility (Myrin Goniometer and according to Suni et al. 1996)	a) NO	GLMM	Mean (SD) Change (95% CI)	a) 0.05 b) 0.2
Headache Impact Test-6 score (HIT, points)	a) NO	GLMM	Mean (SD) Change (95% CI)	a) 0.05 b) 0.2
Neck Disability Index	a) NO	GLMM	Mean (SD) Change (95% CI)	a) 0.05 b) 0.2
Nordic Musculoskeletal Questionnaire	a) NO	GLMM	Mean (SD) Change (95% CI)	a) 0.05 b) 0.2
Fear Avoidance Beliefs Questionnaire	a) NO	GLMM	Mean (SD) Change (95% CI)	a) 0.05 b) 0.2
Work Ability Index	a) NO	GLMM	Mean (SD) Change (95% CI)	a) 0.05 b) 0.2
Quality of life (RAND36)	a) NO	GLMM	Mean (SD) Change (95% CI)	a) 0.05 b) 0.2
Fatigue Impact Scale	a) NO	GLMM	Mean (SD) Change (95% CI)	a) 0.05 b) 0.2
Beck Depression Inventory (BDI)	a) NO	GLMM	Mean (SD) Change (95% CI)	a) 0.05 b) 0.2
Pharmacy claims for headache (euros)	a) NO	GLMM	Mean (SD) Change (95% CI)	a) 0.05 b) 0.2

* From a cohort of Ylinen et al. 2003
** Benjamini & Hoecberg FDR (False Discovery Rate)

4. Figures taken from the blinded data analyses

Figure 1. Headache intensity in Visual Analogue Scale (VAS, absolute numbers per week at baseline, after 3 months and in the end of the 6-month intervention), crude values.

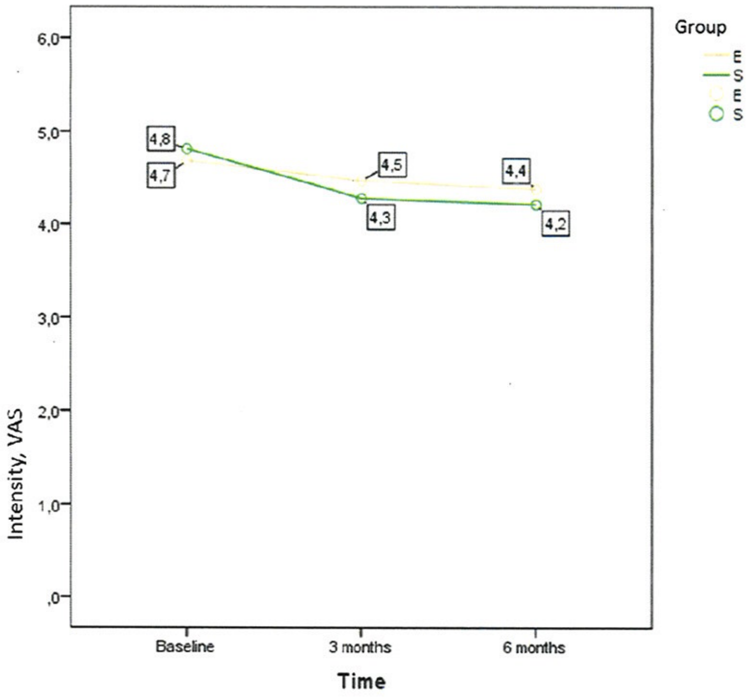

Figure 2. Headache intensity in VAS (per week at baseline, after 3 months and in the end of the 6-month intervention), the unadjusted model.

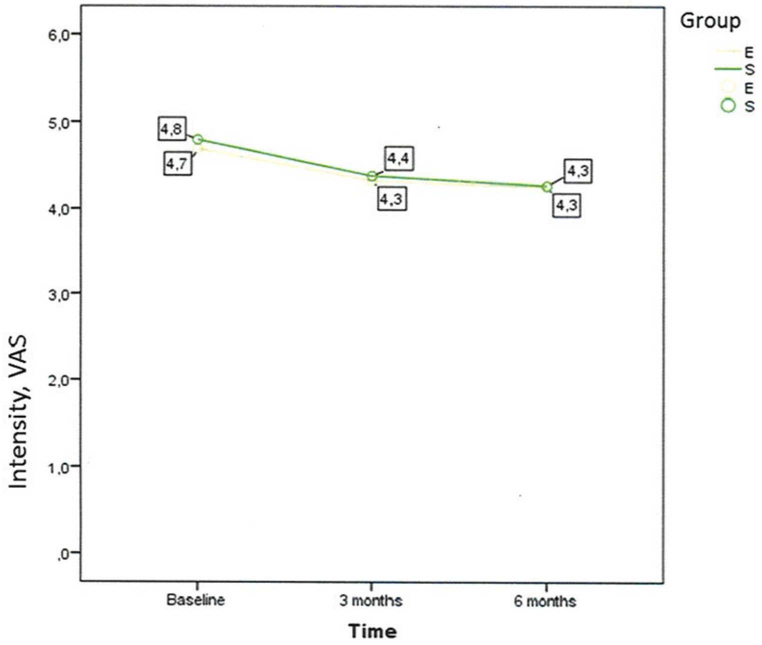

Intensity of headache, VAS

Model Term	Coefficient ▼	Std.Error	t	Sig.	95% Confidence Interval	
					Lower	Upper
**Intercept**	1,692	0,108	15,689	,000	1,480	1,904
Group=2	0,030	0,068	0,442	,659	-0,105	0,165
Group=1	0[a]					
Time	-0,178	0,127	-1,399	,163	-0,429	0,072
Time 2	0,032	0,031	1,032	,303	-0,029	0,094
Time* (group=2)	-0,008	0,039	-0,219	,827	-0,084	0,067
Time* (group=1)	0[a]					

Probability distribution:Gamma
Link function:Log

[a]This coefficient is set to zero because it is redundant.

Figure 3. Headache intensity in VAS (per week at baseline, after 3 months and in the end of the 6-month intervention) adjusted by age, smoking (no vs. yes), and type of work (office vs. other).

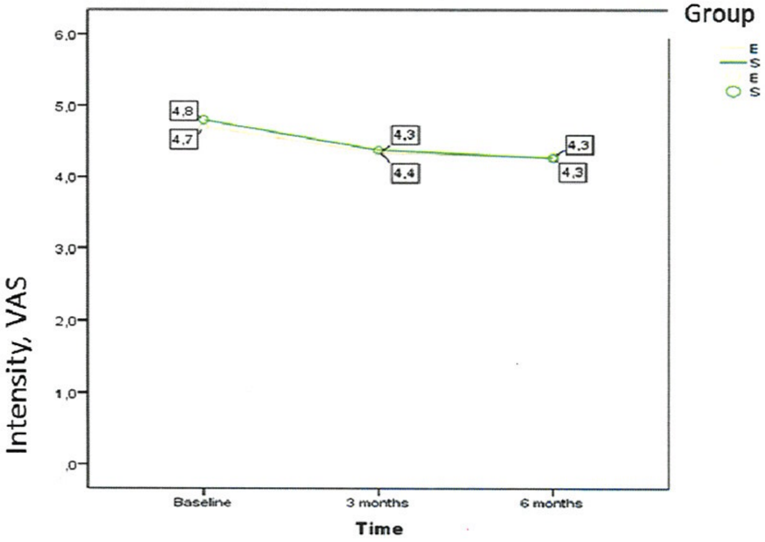

Intensity of headache, VAS

Model Term	Coefficient	Std.Error	t	Sig.	95% Confidence Interval Lower	Upper
Intercept	1,852	0,166	11,141	,000	1,525	2,179
Age	-0,003	0,003	-0,965	,335	-0,008	0,003
Smoking2=1	0,036	0,080	0,448	,655	-0,121	0,193
Smoking2=0	0[a]					
Office W=1	-0,079	0,052	-1,530	,127	-0,180	0,023
Office W=0	0[a]					
Group=2	0,030	0,069	0,429	,668	-0,106	0,166
Group=1	0[a]					
Time	-0,180	0,127	-1,411	,159	-0,430	0,071
Time 2	0,033	0,031	1,063	,289	-0,028	0,095
Time* (group=2)	0,012	0,039	-0,296	,768	-0,088	0,065
Time* (group=1)	0[a]					

Probability distribution: Gamma
Link function: Log

[a]This coefficient is set to zero because it is redundant.

Figure 4. Headache intensity in VAS (per week at baseline, after 3 months and in the end of the 6-month intervention) adjusted by age, smoking, type of work, use of medication related to headache (weekly frequency), work ability index, migraine vs. cervicogenic headache, menstruation status (normal, irregular or perimenopausal/postmenopausal), and hormone therapy (no vs. yes).

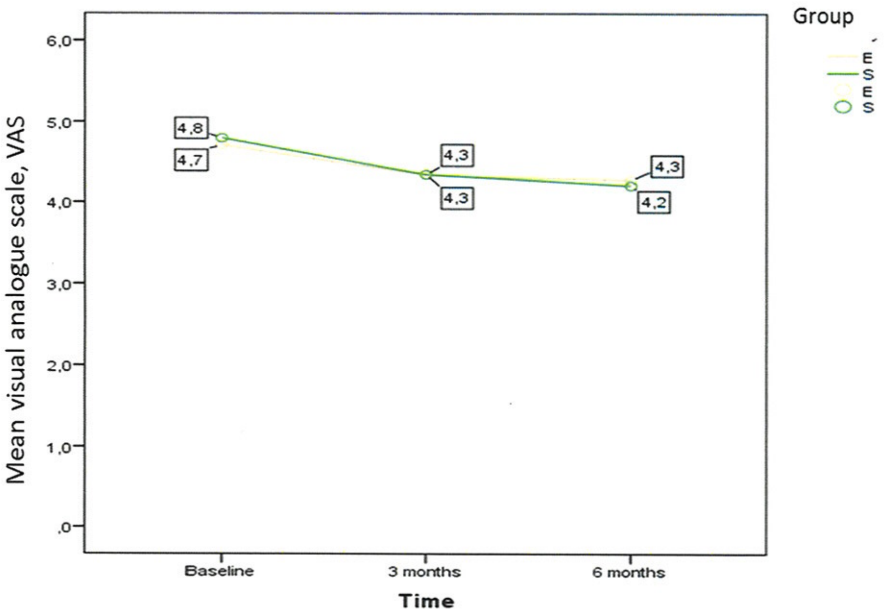

Mean visual analogue scale, VAS

Model Term	Coefficient ▼	Std.Error	t	Sig.	95% Confidence Interval	
					Lower	Upper
Intercept	1,517	0,245	6,186	,000	1,034	1,999
Age	-0,003	0,003	-1,000	,318	-0,010	0,003
Smoking2=1	0,045	0,079	0,572	,568	-0,110	0,200
Smoking2=0	0ª					
Office W=1	-0,077	0,051	-1,502	,134	-0,178	0,024
Office W=0	0ª					
Medication	0,002	0,007	0,320	,749	-0,011	0,015
WorkAbility	0,039	0,019	2,037	,043	0,001	0,076
Headache 2	0,121	0,050	2,397	,017	0,022	0,219
Headache 1	0ª					
HormSta=2	-0,076	0,115	-0,656	,512	-0,303	0,151
HormSta=1	-0,016	0,060	-0,271	,786	-0,135	0,103
HormSta=0	0ª					
Group=2	0,031	0,070	0,442	,659	-0,107	0,168
Group=1	0ª					
Time	-0,179	0,127	-1,412	,159	-0,429	0,071
Time 2	0,033	0,031	1,056	,292	-0,028	0,094
Time* (group=2)	-0,017	0,039	-0,448	,655	-0,094	0,059
Time* (group=1)	0ª					

Probability distribution:Gamma
Link function:Log

ªThis coefficient is set to zero because it is redundant.

Figure 5. Headache frequency per week at baseline, after 3 months and in the end of the 6-month intervention (crude values).

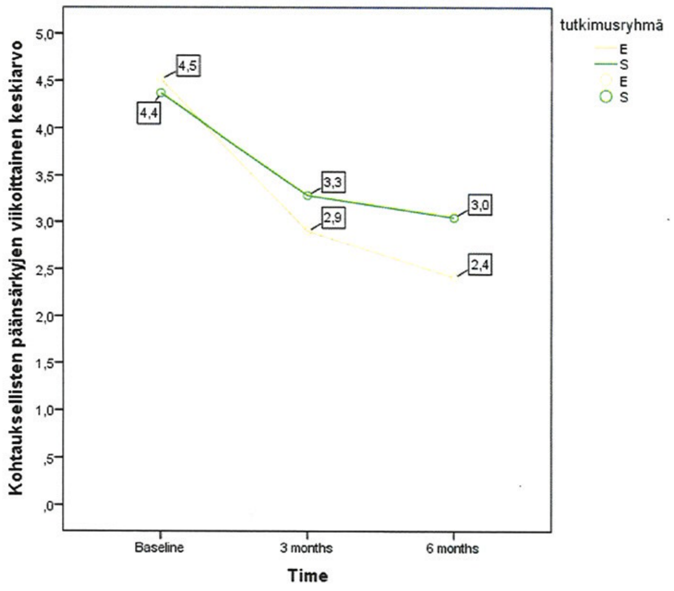

Figure 6. Headache frequency per week at baseline, after 3 months and in the end of the 6-month intervention (the unadjusted model).

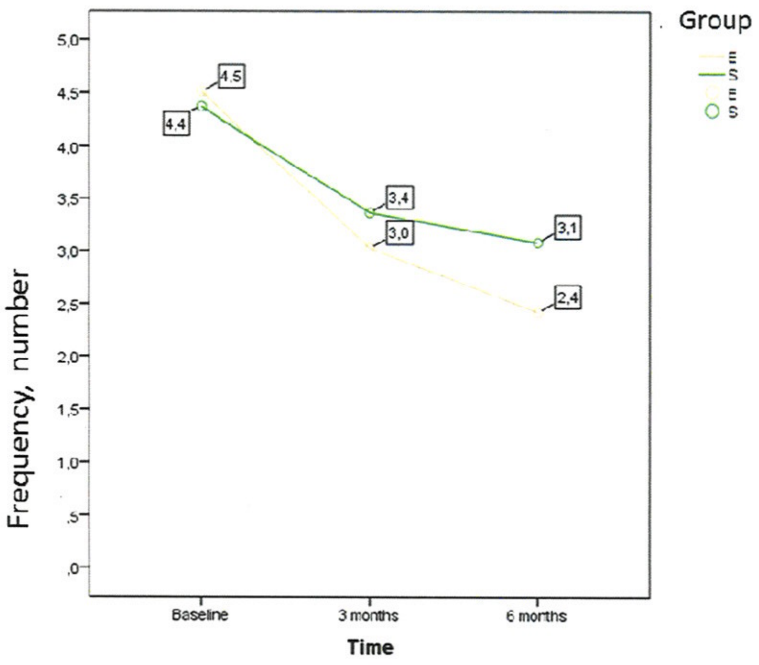

Frequency of weekly headaches, number

Model Term	Coefficient ▼	Std.Error	t	Sig.	95% Confidence Interval	
					Lower	Upper
Intercept	2,080	0,163	12,728	,000	1,758	2,401
Group=2	-0.168	0.141	-1.189	,236	-0.446	0.110
Group=1	0[a]					
Time	-0,661	0,184	-3,596	,000	-1,023	-0,299
Time 2	0,088	0,045	1,953	,052	-0,001	0,176
Time* (group=2)	0,137	0,066	2,087	,038	0,008	0,266
Time* (group=1)	0[a]					

Probability distribution Gamma
Link function:Log

[a]This coefficient is set to zero because it is redundant.

Figure 7. Headache frequency per week at baseline, after 3 months and in the end of the 6-month intervention adjusted by age, smoking (no vs. yes), and type of work (office vs. other).

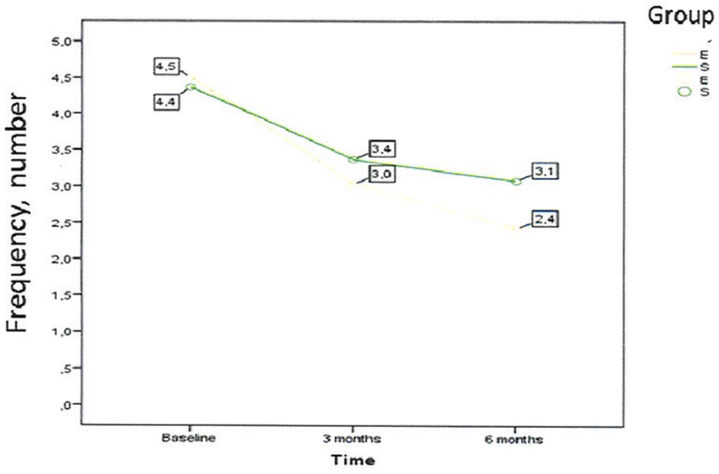

Frequency of weekly headaches, number

Model Term	Coefficient ▼	Std.Error	t	Sig.	95% Confidence Interval Lower	Upper
**Intercept**	2,217	0,317	6,995	,000	1,594	2,841
Age	-0,002	0,006	-0,337	,736	-0,014	0,010
Smoking2=1	-0,128	0,170	-0,756	,450	-0,462	0,206
Smoking2=0	0ª					
Office W=1	-0,068	0,109	-0,620	,536	-0,282	0,147
Office W=0	0ª					
Group=2	-0,174	0,142	-1,226	,221	-0,453	0,105
Group=1	0ª					
Time	-0,654	0,187	-3,505	,001	-1,022	-0,287
Time 2	0,086	0,046	1,879	,061	-0,004	0,175
Time* (group=2)	0,138	0,066	2,089	,038	0,008	0,267
Time* (group=1)	0ª					

Probability distribution:Gamma
Link function:Log

ªThis coefficient is set to zero because it is redundant.

Figure 8. Headache frequency per week at baseline, after 3 months and in the end of the 6-month intervention adjusted by age, smoking, type of work, use of medication related to headache (weekly frequency), work ability index, migraine vs. cervicogenic headache, menstruation status (normal, irregular or perimenopausal/postmenopausal), and hormone therapy (no vs. yes).

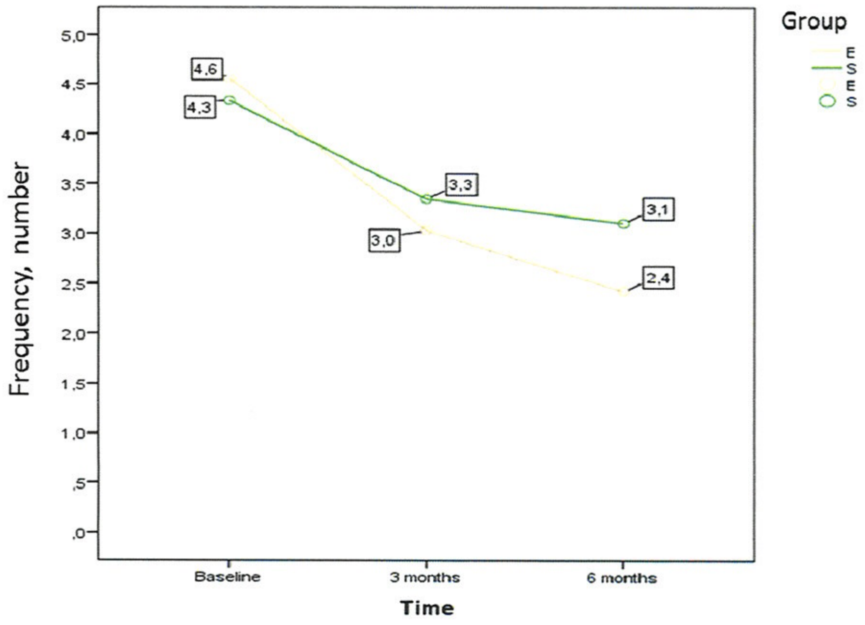

Frequency of weekly headaches, number

Model Term	Coefficient ▼	Std.Error	t	Sig.	95% Confidence Interval	
					Lower	Upper
**Intercept**	3,295	0,414	7,951	,000	2,479	4,110
Age	-0,005	0,006	-0,852	,395	-0,016	0,006
Smoking2=1	-0,127	0,137	-0,925	,356	-0,398	0,143
Smoking2=0	0ª					
Office W=1	0,038	0,089	0,430	,668	-0,138	0,215
Office W=0	0ª					
Medication	0,041	0,012	3,591	,000	0,019	0,064
WorkAbility	-0,129	0,033	-3,896	,000	-0,195	-0,064
Headache 2	-0,310	0,088	-3,535	,000	-0,482	-0,137
Headache 1	0ª					
HormSta=2	0,078	0,200	0,388	,698	-0,316	0,471
HormSta=1	-0,025	0,105	-0,239	,812	-0,232	0,182
HormSta=0	0ª					
Group=2	-0,237	0,114	-2,084	,038	-0,461	-0,013
Group=1	0ª					
Time	-0,683	0,188	-3,624	,000	-1,054	-0,312
Time 2	0,092	0,047	1,965	,050	-0,000	0,183
Time* (group=2)	0,150	0,062	2,411	,017	0,028	0,272
Time* (group=1)	0ª					

Probability distribution:Gamma
Link function:Log

ªThis coefficient is set to zero because it is redundant.

Figure 9. Headache duration (hours per week at baseline, after 3 months and in the end of the 6-month intervention), crude values.

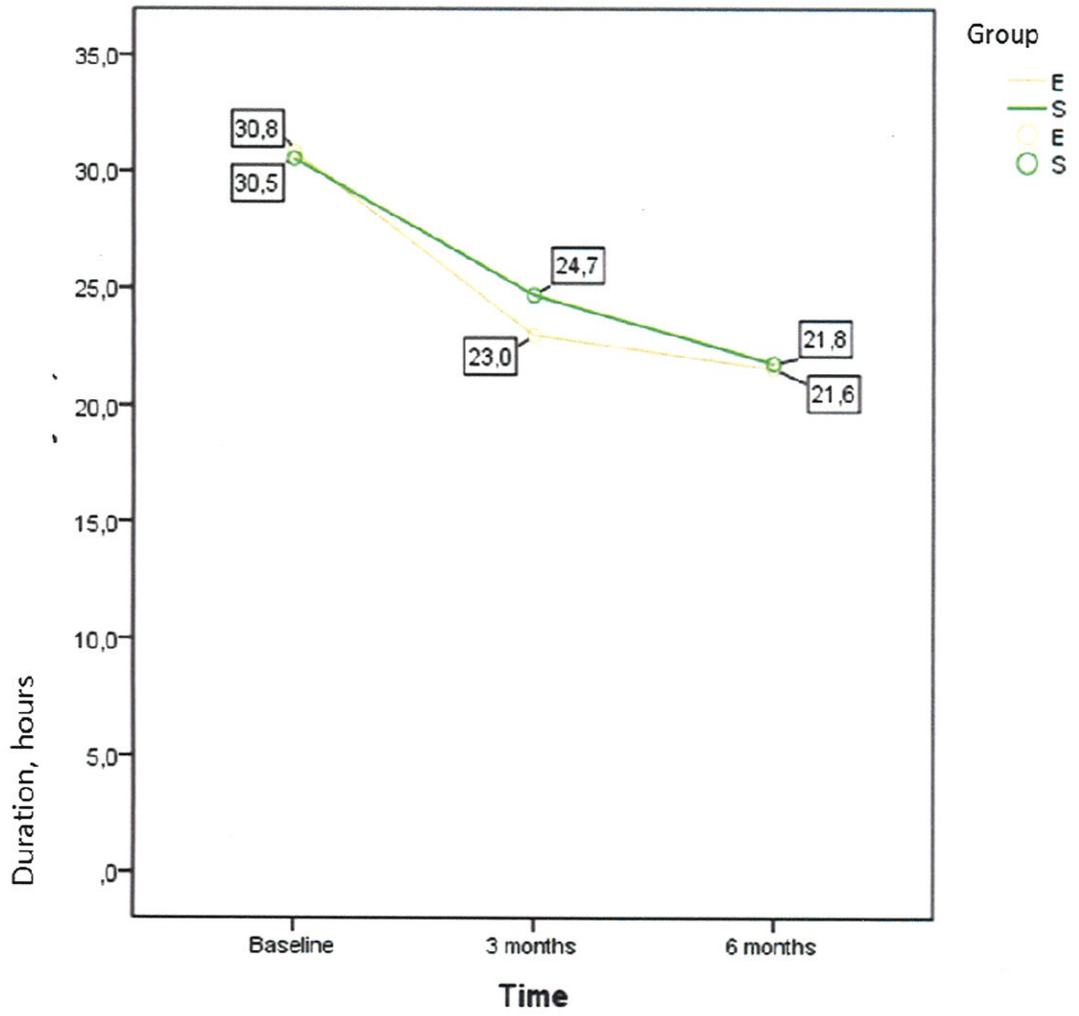

Figure 10. Headache duration (hours per week at baseline, after 3 months and in the end of the 6-month intervention), the unadjusted model.

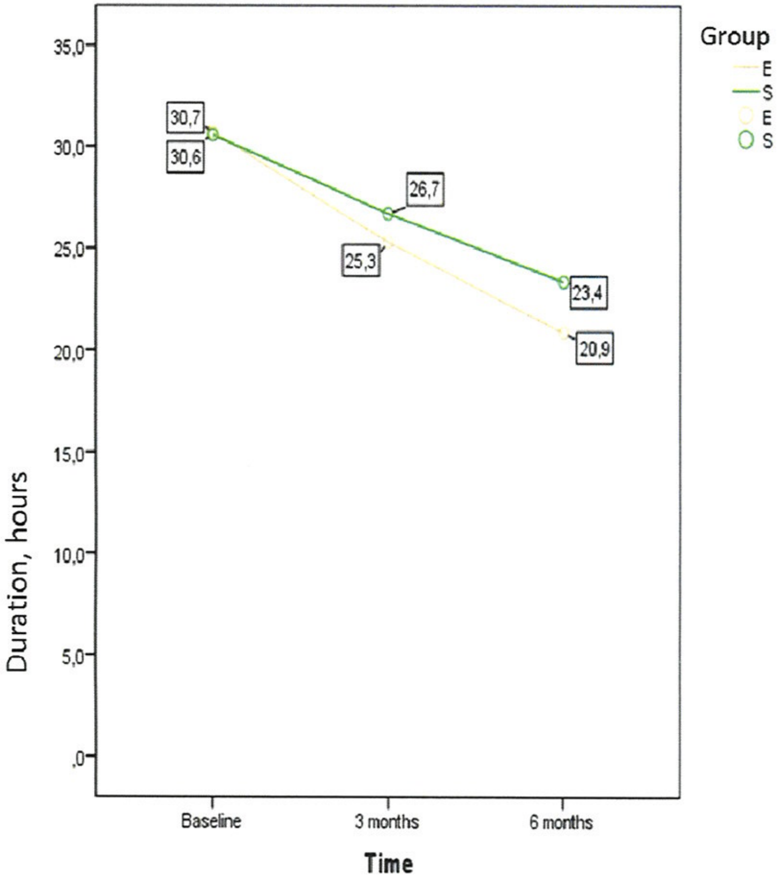

Duration of weekly headaches, hours

Model Term	Coefficient ▼	Std.Error	t	Sig.	95% Confidence Interval	
					Lower	Upper
**Intercept**	3,617	0,149	24,279	,000	3,324	3,910
Group=2	-0,063	0,208	-0,304	,761	-0,473	0,346
Group=1	0[a]					
Time	-0,193	0,073	-2,646	,009	-0,337	-0,050
Time* (group=2)	0,059	0,101	0,584	,560	-0,140	0,258
Time* (group=1)	0[a]					

Probability distribution: Gamma
Link function: Log

[a]This coefficient is set to zero because it is redundant.

Figure 11. Headache duration (hours per week at baseline, after 3 months and in the end of the 6-month intervention) adjusted by age, smoking (no vs. yes), and type of work (office vs. other).

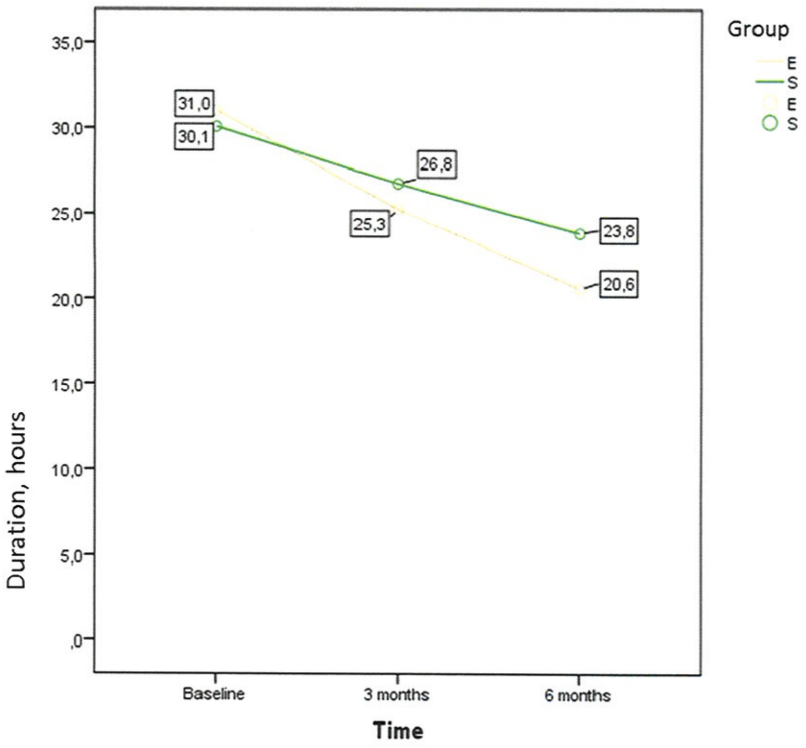

Duration of weekly headaches, hours

Model Term	Coefficient ▼	Std.Error	t	Sig.	95% Confidence Interval	
					Lower	Upper
Intercept	4,301	0,371	11,581	,000	3,570	5,032
Age	-0,016	0,007	-2,103	,036	-0,030	-0,001
Smoking2=1	-0,406	0,213	-1,906	,058	-0,826	0,013
Smoking2=0	0[a]					
Office W=1	0,156	0,137	1,136	,257	-0,114	0,426
Office W=0	0[a]					
Group=2	-0,190	0,214	-0,890	,374	-0,610	0,230
Group=1	0[a]					
Time	-0,206	0,079	-2,612	,009	-0,361	-0,051
Time* (group=2)	0,090	0,109	0,821	,413	-0,125	0,305
Time* (group=1)	0[a]					

Probability distribution: Gamma
Link function: Log

[a]This coefficient is set to zero because it is redundant.

Figure 12. Headache duration (hours per week at baseline, after 3 months and in the end of the 6-month intervention) adjusted by age, smoking, type of work, use of medication related to headache (weekly frequency), work ability index, migraine vs. cervicogenic headache), menstruation status (normal, irregular or perimenopausal/postmenopausal), and hormone therapy (no vs. yes).

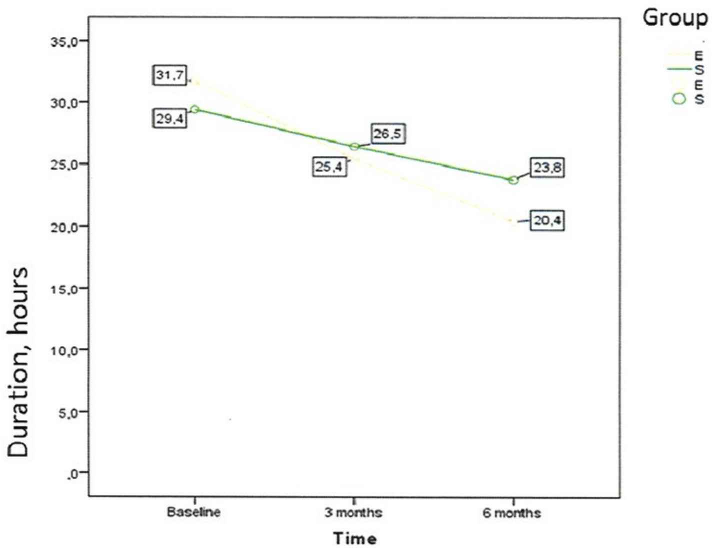

Duration of weekly headaches, hours

Model Term	Coefficient ▼	Std.Error	t	Sig.	95% Confidence Interval Lower	95% Confidence Interval Upper
Intercept	4,558	0,534	8,542	,000	3,508	5,609
Age	-0,012	0,008	-1,588	,113	-0,027	0,003
Smoking2=1	-0,332	0,182	-1,820	,070	-0,691	0,027
Smoking2=0	0[a]					
Office W=1	0,177	0,119	1,490	,137	-0,057	0,410
Office W=0	0[a]					
Medication	0,053	0,015	3,468	,001	0,023	0,083
WorkAbility	-0,073	0,044	-1,653	,099	-0,160	0,014
Headache 2	-0,296	0,117	-2,536	,012	-0,525	-0,066
Headache 1	0[a]					
HormSta=2	0,727	0,270	2,691	,008	0,195	1,259
HormSta=1	0,077	0,141	0,546	,585	-0,200	0,353
HormSta=0	0[a]					
Group=2	-0,275	0,192	-1,434	,153	-0,651	0,102
Group=1	0[a]					
Time	-0,221	0,071	-3,105	,002	-0,361	-0,081
Time* (group=2)	0,115	0,099	1,166	,244	-0,079	0,309
Time* (group=1)	0[a]					

Probability distribution:Gamma
Link function:Log

[a]This coefficient is set to zero because it is redundant.

Figure 13. Quality of life (at baseline and in the end of the 6-month intervention), crude values.

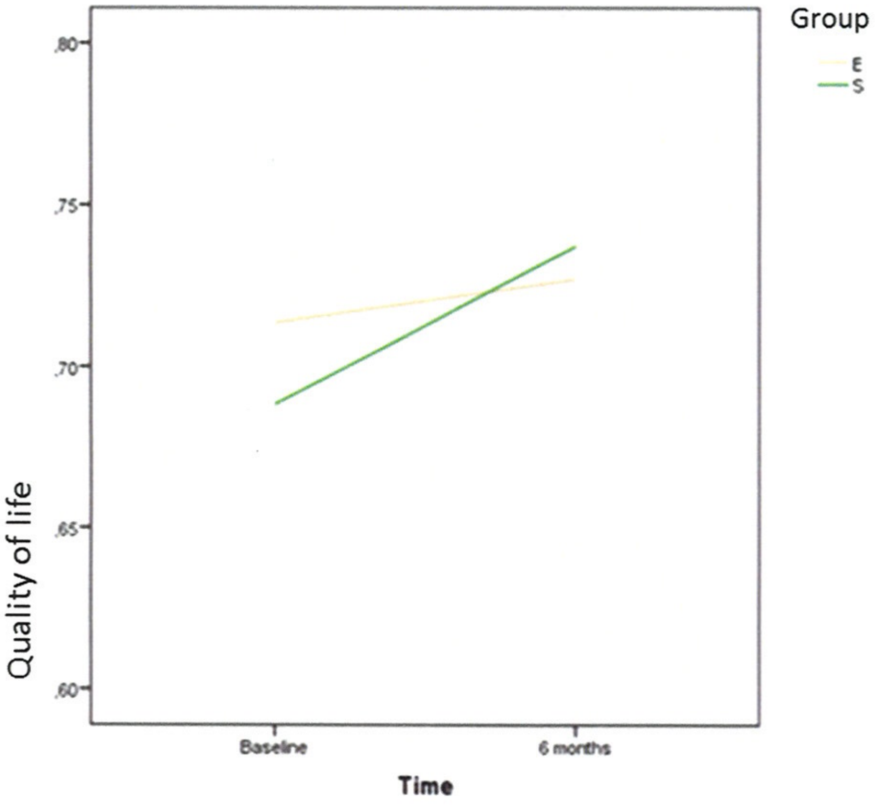

Figure 14. Quality of life (at baseline and in the end of the 6-month intervention) adjusted by age, smoking (no vs. yes), and type of work (office vs. other).

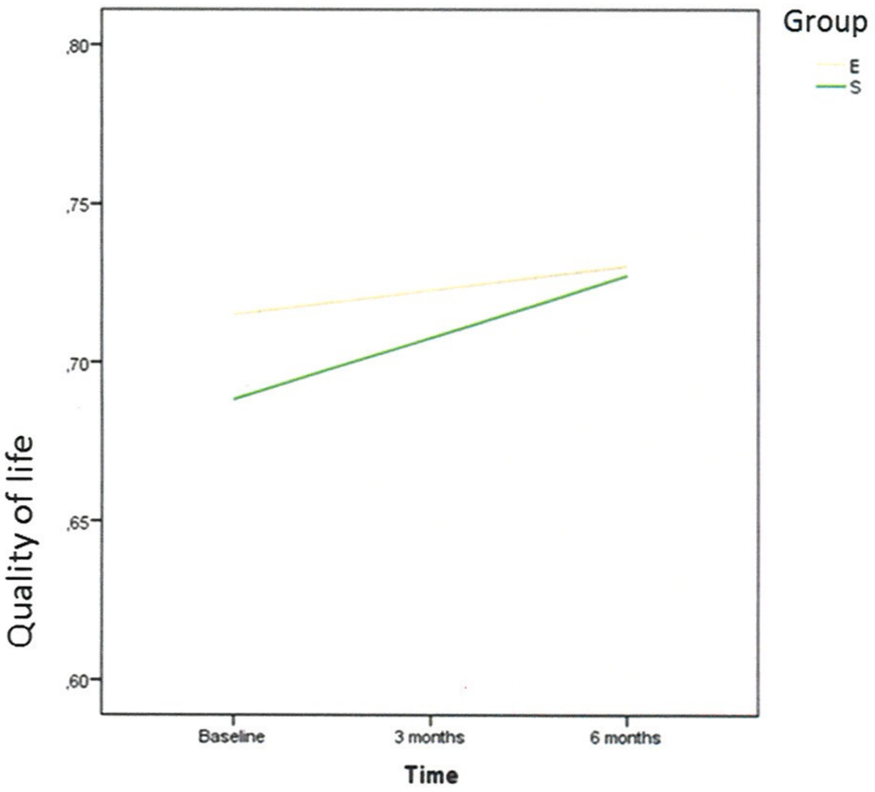

Quality of life

Model Term	Coefficient ▼	Std.Error	t	Sig.	95% Confidence Interval	
					Lower	Upper
Intercept	0,611	0,055	11,169	,000	0,503	0,718
Age	0,001	0,001	0,716	,475	-0,001	0,003
Smoking2=0	0,028	0,030	0,941	,348	-0,030	0,086
Smoking2=1	0[a]					
Office W=0	0,004	0,019	0,232	,817	-0,033	0,042
Office W=1	0[a]					
Group=1	0,037	0,024	1,542	,125	-0,010	0,084
Group=2	0[a]					
Time	0,020	0,008	2,448	,015	0,004	0,036
Time* (group=1)	-0,012	0,011	-1,062	,290	-0,035	0,010
Time* (group=2)	0[a]					

Probability distribution:Normal
Link function:Identity

[a]This coefficient is set to zero because it is redundant.

Figure 15. Use of medication related to headache (at baseline and in the end of the 6-month intervention), crude values.

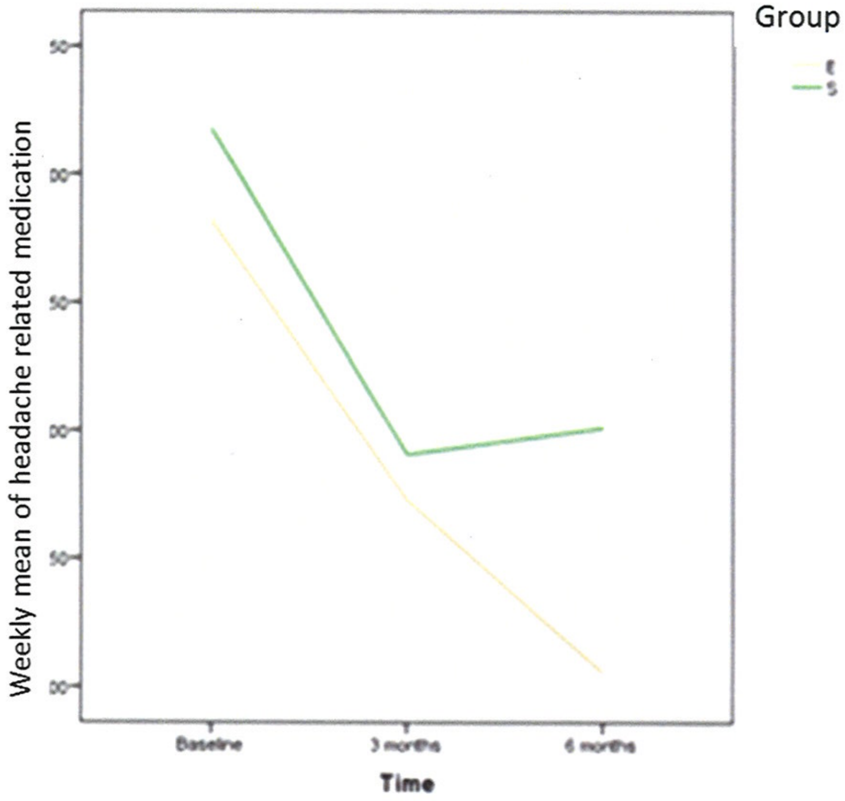

Figure 16. Use of medication related to headache (at baseline and in the end of the 6-month intervention) unadjusted model.

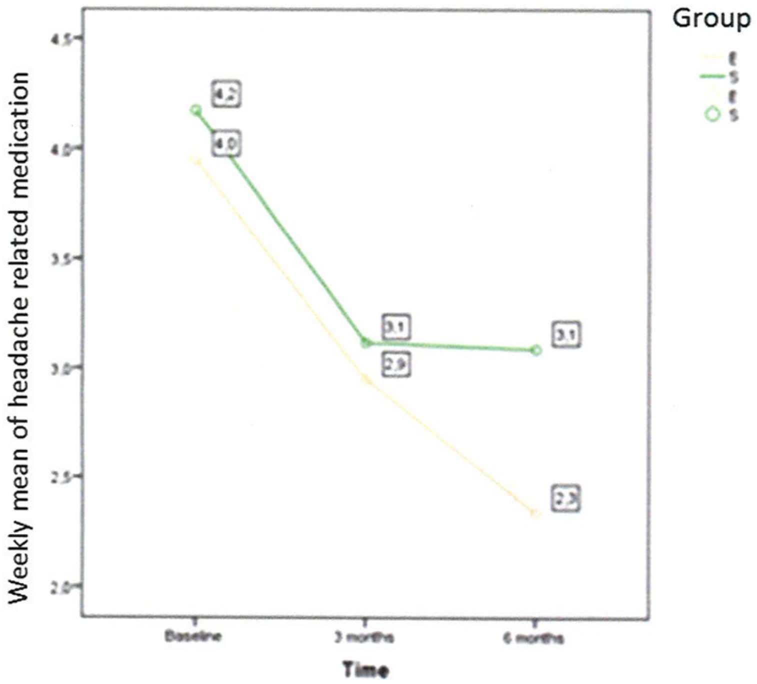

Weekly mean of headache related medication

Model Term	Coefficient ▼	Std.Error	t	Sig.	95% Confidence Interval	
					Lower	Upper
Intercept	1.728	0.415	4.161	.000	0.911	2.546
Group=2	0.277	0.568	0.487	.627	-0.841	1.395
Group=1	0*					
Time	-0.385	0.572	-0.674	.501	-1.510	0.740
Time 2	0.031	0.154	0.199	.842	-0.272	0.334
Time* (group=2)	-0.331	0.782	-0.424	.672	-1.870	1.207
Time* (group=1)	0*					
Time2* (group=2)	0.111	0.210	0.528	.598	-0.302	0.524
Time2* (group=1)	0*					

Probability distribution Gamma
Link function Log

*This coefficient is set to zero because it is redundant
